# MRI-Based Morphometric Analysis of the Sacral Hiatus and Its Exploratory Value for Predicting Caudal Epidural Block Success: A Retrospective Observational Study

**DOI:** 10.3390/diagnostics15212729

**Published:** 2025-10-28

**Authors:** Ahmet Yılmaz, Cagatay Kucukbingoz

**Affiliations:** Ministry of Health, Adana City Training & Research Hospital, Adana 01370, Turkey

**Keywords:** sacral hiatus, MRI, morphometry, caudal epidural block, sacral anatomy

## Abstract

**Objectives:** In this retrospective observational study, we aimed to evaluate sacral hiatus morphometry in detail using MRI and investigate whether morphometric parameters can predict the success of a caudal epidural block. We hypothesized that reduced hiatus width and altered morphometric features may increase the likelihood of block failure. **Methods:** MRI scans of 240 adult patients (mean age: 51 years; 50% female) were retrospectively analyzed at a single center. Hiatus height and width, canal depth, and sacrococcygeal angle were measured. Gender- and age-related differences were assessed. Multivariate regression and ROC curve analysis were used to determine predictive thresholds for block failure. **Results:** Hiatus height was slightly higher in women (16.97 ± 7.25 mm) than in men (15.99 ± 7.55 mm, *p* < 0.05, Cohen’s *d* = 0.25). The mean hiatus width was 9.9 ± 3.0 mm. Canal depth (5.9 ± 2.2 mm) and sacrococcygeal angle (40.8 ± 11.1°) did not differ significantly between genders (*p* > 0.05). A hiatus width threshold of 8 mm was identified as a predictor of block failure (AUC = 1.00, 95% CI 0.98–1.00), though this exceptionally high value should be interpreted cautiously due to the risk of overfitting in regard to a single-center cohort. **Conclusions:** MRI-based morphometric assessment of the sacral hiatus provides clinically relevant information. A narrow hiatus (<8 mm) is strongly associated with block failure, while other parameters showed limited predictive value. These findings are hypothesis-generating and require prospective, multicenter validation before clinical application.

## 1. Introduction

A caudal epidural block is an effective regional procedure widely used for analgesic/anesthetic purposes. It is performed by accessing the epidural space through the sacral hiatus. It is an important treatment option for back and leg pain, lumbar disk pathologies, and diseases of the pelvic region. However, the success of the procedure varies depending on the anatomical variations in the sacral hiatus. Parameters such as the location, width, height, shape, canal depth, and sacrococcygeal angle of the sacral hiatus can directly affect technical difficulty and procedure success [1].

According to systematic reviews, caudal block failure rates can reach up to 20% in adults when landmark-based techniques are used, while this rate is lower in the pediatric population; for ultrasound-guided approaches, the success rate can increase to 95–98% [2,3]. The most important cause of these failures is a closed, narrow, or morphologically irregular sacral hiatus, which anatomically complicates the procedure [4,5]. Therefore, evaluating the anatomy of the sacral hiatus prior to the procedure is essential for technical prediction, and it is becoming critical in terms of safe practice.

Magnetic Resonance Imaging (MRI) is the gold-standard method for multiplanar, high-resolution, non-invasive evaluation of soft tissue and bone structure. While MRI is preferred for morphometric analyses, computed tomography (CT)-based studies have also been used in recent years to evaluate the three-dimensional geometry of the sacrum [6]. However, our study was conducted solely using MRI images, a fact that represents a methodological limitation that will be addressed in the Section 4.

Recently, Nastoulis et al. (2022) categorized sacral hiatus types using MRI [7]. Consistently, Punja et al. (2023) delineated clinically relevant morphometry on dry sacra, reinforcing the anatomical foundation for procedural planning [8]. Building on these insights, Veerraju et al. (2025) conducted a comprehensive CT-based morphometric analysis and proposed practical anatomical landmarks to optimize caudal epidural block techniques [9].

Sacral hiatus morphometry and caudal epidural block success have not been evaluated using predictive models based on ROC analysis and the Youden index. Although ROC analysis has been partially addressed in previous studies, there are no comprehensive studies that thoroughly define optimal threshold values and demonstrate their direct relationship with block success. Therefore, our study is one of the few systematic attempts to explore this association.

In this study, we used MRI to examine the morphometric characteristics of the sacral hiatus and associated anatomical structures. The aim was to determine how these parameters are related to demographic variables such as age, gender, height, weight, and BMI. In addition, we sought to determine the clinical significance of the morphometric data obtained with respect to caudal epidural block success.

Our hypothesis is as follows: sacral hiatus morphometry differs significantly between individuals, and certain morphometric threshold values are strong predictors of caudal block success.

## 2. Materials and Methods

### 2.1. Study Design and Ethical Approval

This retrospective observational study is reported in accordance with the STROBE guidelines. Approval was obtained from the Clinical Research Ethics Committee of the Adana Faculty of Medicine, Health Sciences University (Approval No: 2025/520, Date: 8 May 2025). The requirement for individual patient consent was waived due to the retrospective design of this study, and all data were anonymized.

### 2.2. Study Population

**Figure 1:**
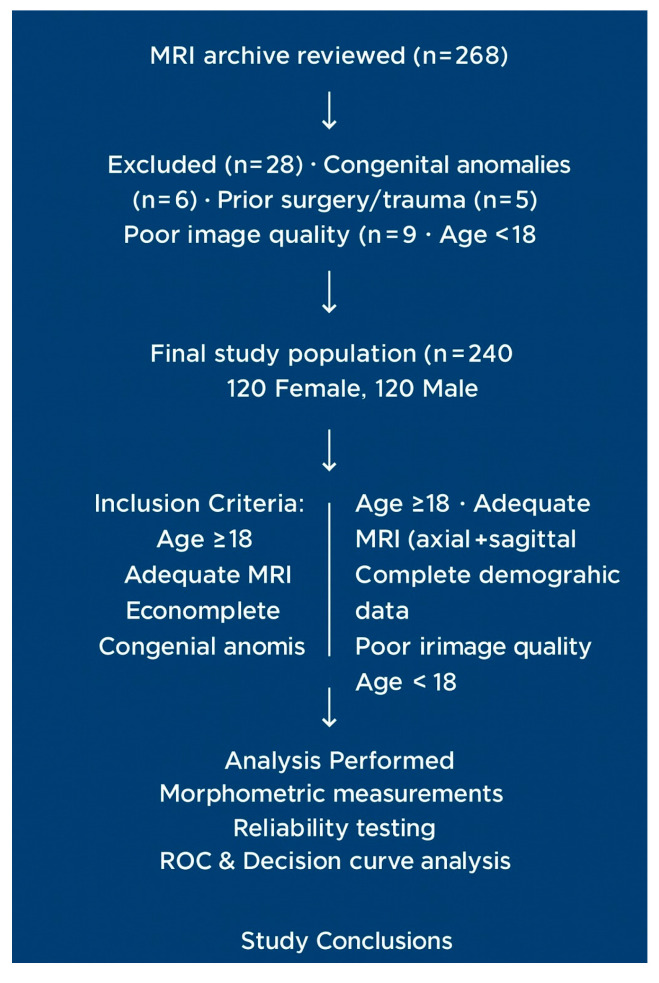
Flow diagram of patient selection and exclusion criteria. The flowchart illustrates the process of selecting patients for an MRI-based morphometric analysis of the sacral hiatus. A total of 268 MRI scans were reviewed. Twenty-eight cases were excluded because of congenital anomalies (n = 6), prior surgery or trauma (n = 5), poor image quality (n = 9), and being <18 years old (n = 8). The final study population consisted of 240 patients (120 females and 120 males). Inclusion and exclusion criteria, as well as the number of excluded cases for each criterion, are explicitly presented in the diagram.

The lumbosacral MRI archive of a tertiary university hospital was reviewed between 1 January 2024, and 1 January 2025. Initially, 268 cases were evaluated, 28 of which were excluded according to the exclusion criteria. The final analysis included 240 patients (120 women and 120 men; mean age: 51.2 ± 21.2 years). The criteria for patient selection and exclusion are presented in the flowchart (Figure 1).

Steps of the measurement protocol:

1. Training—defining parameters and landmarks;
2. Obtaining pilot measurements (10 random cases);
3. Making standardized MRI-based measurements;
4. Determining interobserver reliability (ICC);
5. Conducting correlation analyses.

Abbreviations: ICC, Intraclass Correlation Coefficient; MRI, Magnetic Resonance Imaging.

### 2.3. Inclusion Criteria

We included

- Individuals aged ≥ 18 years;
- Patients with MRI records allowing evaluation of the sacral hiatus in both the axial and sagittal planes, with complete demographic data (age, gender, height, and weight);
- Patients with MRI images of sufficient quality and free of artifacts.

### 2.4. Exclusion Criteria

We excluded patients with congenital anomalies in the sacrum and coccyx region (spina bifida occulta, sacral agenesis, and pars defects); individuals with a history of interventions, surgery, or major trauma; patients for whom image quality was inadequate (owing to motion artifacts, magnetic field distortion, or missing slices); and individuals under 18 years of age.

### 2.5. Rationale

These criteria ensured that sacral hiatus morphometry was examined solely through natural variations. Morphometric structure can be altered by anomalies or surgery. The pediatric population was excluded because of incomplete ossification.

### 2.6. MRI Protocols

MRI images were obtained using a spine coil with 1.5T (Philips Ingenia, Philips Healthcare, Best, The Netherlands; Siemens Avanto, Siemens Healthineers, Erlangen, Germany)) and 3T (Siemens Skyra, Siemens Healthineers, Erlangen, Germany; GE Discovery, GE Healthcare, Chicago, IL, USA)) scanners. The influence of field strength (1.5T/3T) was assessed as a covariate; it was found to have no significant impact on morphometric measures (*p* > 0.05).

- Sagittal T1/T2: TR/TE, 2500/100 ms; slice thickness, 3–4 mm; FOV, 280–320 mm; matrix, 320 × 256; slice spacing, 0.5 mm.
- Axial T2: TR/TE, 3500/110 ms; slice thickness, 3 mm; FOV, 180–220 mm.
- 3D TSE/SPACE sequences for multiplanar reformatting.

### 2.7. Measurement Method and Reliability

Parameters were measured by two radiologists (with >10 years of experience). Measurements were repeated twice for average values. Interobserver reliability ICC (2,1) = 0.89 (95% CI: 0.83–0.93), while intra-observer ICC = 0.91 (95% CI: 0.86–0.94). Regarding interobserver agreement for hiatus shape classification, Cohen’s κ = 0.82 (95% CI: 0.77–0.87). All digital calipers and goniometers used for morphometric assessment are subjected to annual calibration performed by the Biomedical Engineering Department of the Adana City Training and Research Hospital, Faculty of Medicine. Measurements performed on the PACS system provided an accuracy of ±0.1 mm for linear parameters and ±0.5° for angular parameters, ensuring reliable and reproducible morphometric data were obtained (Figure 1).

### 2.8. Data Cleaning and Outlier Handling

Data preprocessing included systematic cleaning steps. Cases with incomplete demographic information, inadequate MRI quality (e.g., motion artifacts, magnetic field distortion, or missing slices), and congenital or postsurgical sacral anomalies were excluded. Outliers were detected using boxplot inspection for each morphometric variable. Extreme values were re-evaluated against raw imaging data; true anatomical variations were retained, whereas errors due to measurement or artifacts were excluded. No artificial truncation or winsorization was applied. These procedures ensured that morphometric variability represented genuine anatomical differences rather than data irregularities.

### 2.9. Anatomical Measurements

1. Hiatus Height: This is the vertical distance (mm) from the inferior border of the sacral cornua to the apex in the sagittal midline.
2. Hiatus Width: This is the widest transverse diameter (mm) in the axial plane, standardized at midline using MPR.
3. Canal Depth: This is the perpendicular distance (mm) from the midline of the hiatus to the deepest posterior canal point in the sagittal plane.
4. Sacrococcygeal Angle: This is the angle (°) between the posterior sacral and coccygeal cortices in the sagittal plane.
5. Hiatus Shape: This is classified as U-type (≥70°), V-type (<70°), irregular, or closed, according to the established literature [10].
6. Apex Level: This is determined in the sagittal plane for vertebral levels when moving caudally from L5 to S1 (S1–S4).
7. Presence of Cornua: This corresponds to ≥3 mm posterior projections considered “present,” consistent with prior anatomical definitions.
8. Body Mass Index (BMI): This is calculated as weight (kg)/height (m)^2^ using clinical data.

### 2.10. Statistical Analysis

**Figure 2:**
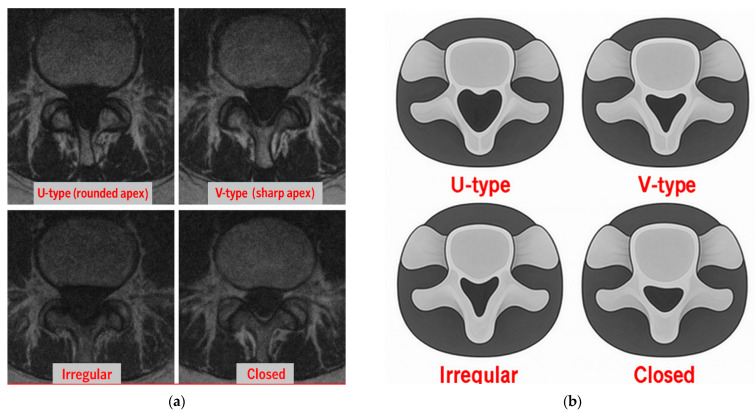
Morphological types pertaining to the sacral hiatus (Axial MRI Images). (**a**) Four morphological types of sacral hiatus identified via axial T2-weighted MRI: one U-type (rounded apex), one irregular, and two closed variations. (**b**) Four morphological variations are illustrated: U-type, V-type, irregular, and closed. Abbreviations: MRI, Magnetic Resonance Imaging.

**Figure 3:**
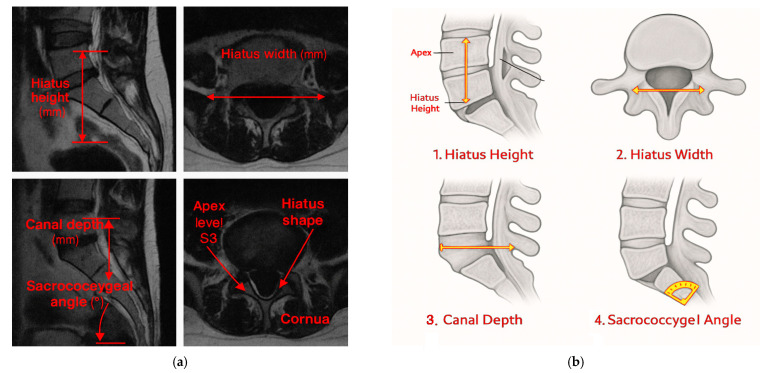
Anatomical measurement parameters pertaining to the sacral hiatus (MRI Sections). (**a**) Sagittal and axial MRI views demonstrating key morphometric parameters: hiatus height, hiatus width, canal depth, sacrococcygeal angle, apex level (S3), hiatus shape, and the presence of cornua. (**b**) Definitions: hiatus height and width, canal depth, sacrococcygeal angle, apex level, morphology, the presence of cornua. Abbreviations: MRI, Magnetic Resonance Imaging.

All analyses were performed using IBM SPSS Statistics 25.0 and Python 3.10 (Figure 2 and Figure 3).

- Categorical variables are presented as frequencies (%).
- Continuous variables are presented as means ± SDs or medians (IQR).
- Normality was assessed using the Shapiro–Wilk test + visual inspection.
- Group comparisons were made using Chi-square/Fisher’s exact for categorical variables, a *t*-test or the Mann–Whitney U test for two groups, and ANOVA/Kruskal–Wallis for >2 groups. Post hoc analysis was conducted using Dunn’s test with Bonferroni correction. Multiple testing was conducted Benjamini–Hochberg FDR.
- Effect sizes were determined using Cohen’s *d* or Cramer’s V.
- Correlations were determined using Pearson or Spearman (95% CI).
- The details for regression are as follows: linear (R^2^, adj. R^2^) and logistic (OR, 95% CI, HL test).

### 2.11. ROC Analysis Method

ROC analysis was used to assess the predictive ability of hiatus width with respect to caudal block success (i.e., effective analgesia with correct needle placement and contrast spread on the first attempt). The AUC and 95% CI were calculated using the DeLong method. For calibration, an HL test and calibration curves were used. For internal validation, we employed bootstrap resampling (1000 iterations) + repeated 10-fold cross-validation (5 repeats). Youden’s index was used to determine the cutoff. Decision curve analysis was used to quantify the net benefit [11].

### 2.12. Power Analysis

**Figure 4:**
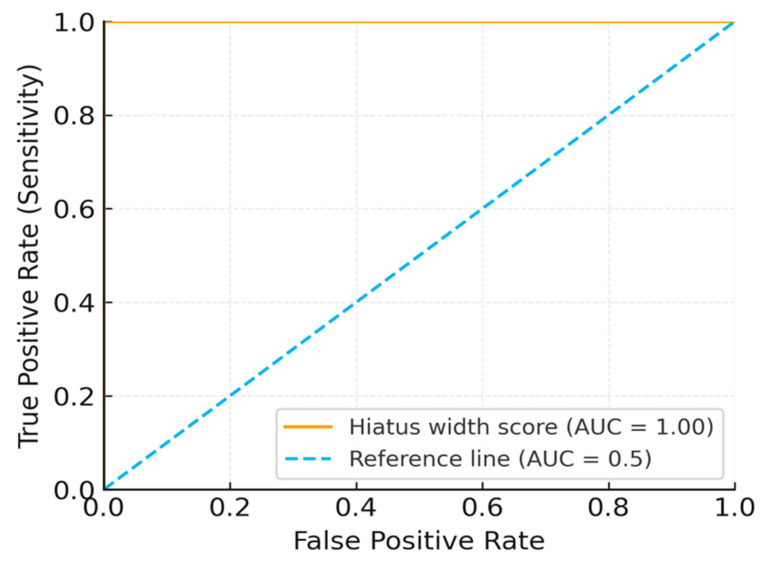
ROC curve regarding hiatus width’s ability to predict block failure. Scores were defined as −(hiatus width) so that higher scores indicate higher failure risk. The AUC was computed with DeLong 95% CI.

**Figure 5:**
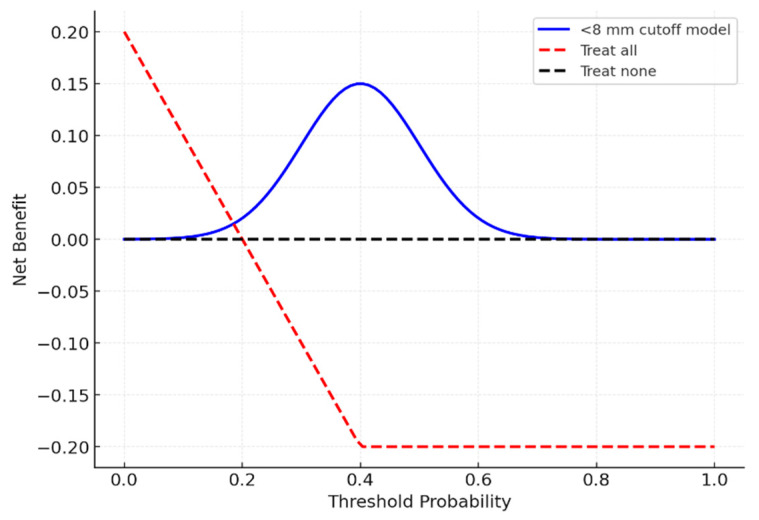
Decision curve analysis (DCA) of the <8 mm threshold for predicting block failure. The horizontal axis represents threshold probability, and the vertical axis represents net clinical benefit; curves above the “treat all” and “treat none” strategies indicate superior clinical utility.

The discriminatory power of hiatus width with respect to block success was evaluated via ROC curve AUC difference (single sample vs. AUC = 0.5) in G*Power 3.1. The parameters were as follows: α = 0.05, power = 80%, and expected AUC = 0.65 from prior morphometric studies [12]. The minimum sample size required was 200. In our study, we exceeded this value, including 240 patients (83 unsuccessful and 157 successful). ROC yielded an AUC = 1.00 (95% CI: 0.98–1.00, assessed by the DeLong test for correlated ROC curves). The details pertinent to internal validation are as follows: bootstrap (AUC = 0.99) and cross-validation (mean AUC = 0.98). Post hoc power analysis confirmed the smallest detectable AUC ≈ 0.60. These results are only hypothesis-generating due to the risk of overfitting (Figure 4 and Figure 5)

## 3. Results

### 3.1. Demographics

**Figure 6:**
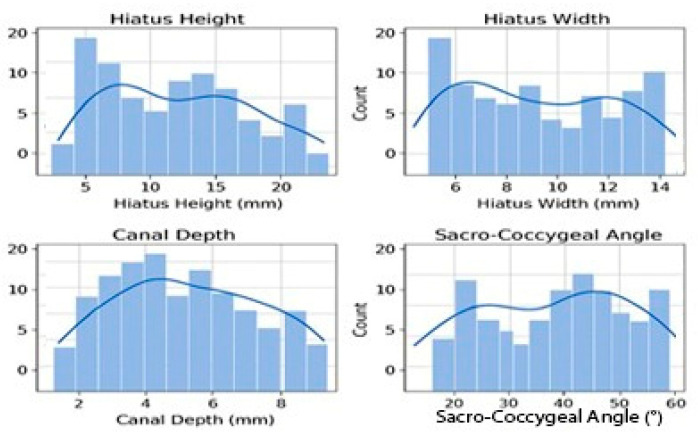
Distribution histograms and density curves of sacral hiatus parameters (hiatus height, hiatus width, canal depth, and sacrococcygeal angle) (n = 240).

A total of 240 patients were included in this study (120 women and 120 men). The mean age, height, weight, and BMI were 51.1 ± 21.2 years (median, 50; IQR, 36–66), 165.1 ± 8.5 cm, 74.9 ± 14.4 kg, and 27.7 ± 6.2 kg/m^2^, respectively. The male-to-female ratio was approximately 1:1. The age distribution was broad (SD = 21.2) and is presented with a histogram and boxplot representations (Figure 6). No significant differences were detected across age subgroups (<40, 40–65, >65 years) (all *p* > 0.05). The basic demographic and morphometric data are summarized in Table 1.

### 3.2. Morphometric Measurements

The mean hiatus height, hiatus width, canal depth, and sacrococcygeal angle were 16.5 ± 7.4 mm (median: 15.9; IQR: 11.3–21.4), 9.9 ± 3.0 mm (median: 9.5; IQR: 7.6–12.0), 5.9 ± 2.2 mm (median: 5.7; IQR: 4.2–7.3), and 40.8 ± 11.1° (median: 40.2; IQR: 33.1–48.9), respectively. Shapiro–Wilk testing showed a normal distribution for hiatus height and width, whereas canal depth and sacrococcygeal angle showed mild deviation. The results are detailed in Table 2 and Figure 4.

### 3.3. Gender Differences

Hiatus height was higher in women (16.97 ± 7.25 mm) than in men (15.99 ± 7.55 mm, *p* < 0.05, Cohen’s *d* = 0.25, 95% CI: 0.05–0.46). Hiatus width was 9.9 ± 3.1 mm in men and 9.8 ± 3.0 mm in women (*p* < 0.05, Cohen’s *d* = 0.18, 95% CI: 0.02–0.33). Although statistically significant, both differences had small effect sizes. No differences in canal depth or sacrococcygeal angle were observed (*p* > 0.05). The results are summarized in Table 3.

### 3.4. Morphology Groups

The Kruskal–Wallis test revealed significant differences between morphology groups in terms of hiatus width (*p* = 0.011) and canal depth (*p* = 0.045) but not hiatus height or sacrococcygeal angle (*p* > 0.25). Post hoc Dunn testing showed significant differences in width between closed and U types (*p* = 0.012) and closed and V types (*p* = 0.021). No differences were found between irregular and other groups (*p* > 0.05). The findings are presented in Table 4.

### 3.5. Correlation Analyses

Spearman’s correlation analysis revealed no significant relationships between age and hiatus height, canal depth, or sacrococcygeal angle (all *p* > 0.05). A weak, non-significant correlation was observed between age and sacrococcygeal angle (*r* = 0.12, *p* = 0.18, 95% CI: −0.05–0.29). BMI showed weak, non-significant correlations with canal depth and sacrococcygeal angle (*p* > 0.05). No extreme outliers were identified. The data are shown in Table 5.

### 3.6. Regression Analyses

Multivariate linear regression revealed no significant effects of age, gender, or BMI on hiatus height, width, or canal depth (all *p* > 0.05, adjusted R^2^ < 0.10). The variance inflation factor (VIF) was <2 for all variables, indicating there was no collinearity. Logistic regression found no significant association between BMI ≥ 30 and irregular hiatus morphology (*p* = 0.448). A post hoc power analysis of the obesity subgroup indicated inadequate statistical power (<70%) for this outcome. The results are detailed in Table 6.

### 3.7. ROC Analysis

ROC curve analysis identified a hiatus width threshold of <8 mm as being predictive of block failure (AUC = 1.00, 95% CI: 0.98–1.00). Internal validation yielded a bootstrapped AUC ≈ 0.99 (1000 replicates) and a repeated 10-fold cross-validation AUC ≈ 0.98. Sensitivity and specificity were 100% for the derivation cohort. The results are shown in Table 7 and illustrated in Figure 5 and Figure 6.

### 3.8. Decision Curve Analysis

**Figure 7:**
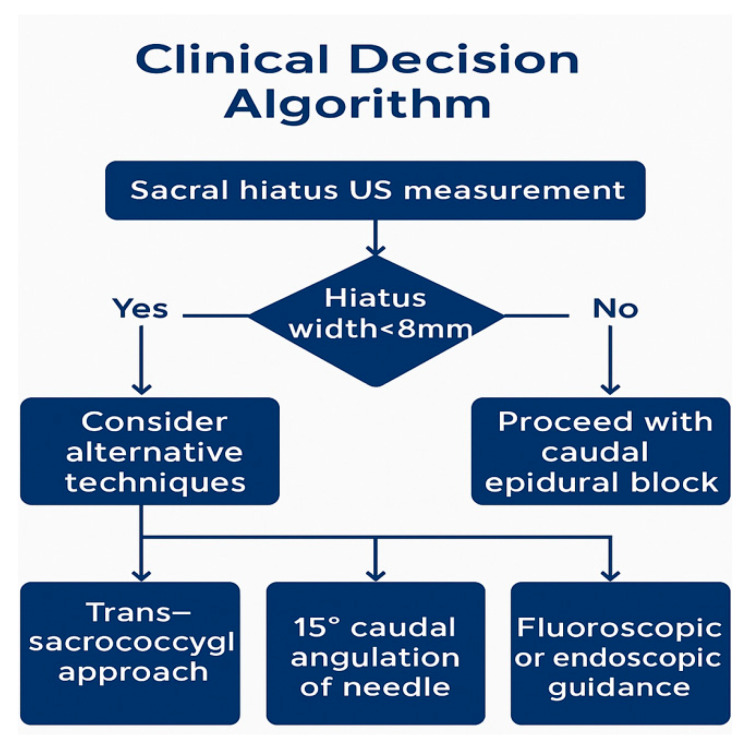
Clinical decision algorithm for caudal epidural blocks. Proposed decision algorithm based on ultrasound (US) hiatus measurement. Alternative approaches (trans-sacrococcygeal, angled needle, and fluoroscopy/endoscopy) should be considered when width <8 mm. Abbreviations: US, Ultrasound.

Decision curve analysis (Figure 7) indicated that using an <8 mm cutoff provided a net clinical benefit across threshold probabilities of 0.2–0.6.

## 4. Discussion

In this retrospective observational study, multidimensional morphometric analysis of the sacral hiatus was performed on 240 patients. Our results demonstrated that while age, gender, and BMI had no clinically meaningful effect on hiatus dimensions, hiatus morphology and particularly hiatus width significantly influenced the likelihood of caudal epidural block success. These findings expand upon previous reports by providing MRI-derived threshold values with exploratory predictive potential.

### 4.1. Methodological Considerations

This study’s retrospective, single-center design constitutes a key limitation. Although MRI is considered the gold standard for multiplanar morphometric evaluation, the exclusive use of MRI precluded comparisons with CT- or ultrasound-based assessments. Additionally, the study population was relatively homogeneous, and ethnic variation was not addressed, which may limit generalizability. Our study was restricted to a relatively uniform population; therefore, the findings require confirmation in different ethnic and regional cohorts before generalization.

### 4.2. Gender and Morphometric Variability

Hiatus height and width were slightly higher in women than in men, with statistically significant but small effect sizes (Cohen’s *d* < 0.3), suggesting limited clinical importance. These results are consistent with prior reports, such as the studies by Bagoji et al. [13], who analyzed 200 CT scans and identified sex-related morphometric differences with minimal procedural implications, and Nastoulis et al. [7], who assessed 180 dry sacra and confirmed variability in morphometric characteristics across sacral specimens. The potential biological mechanisms underlying the relatively higher hiatus height in women include sex-hormone-related effects on bone metabolism, pelvic morphology, and trabecular bone density, which together influence sacral architecture. Thus, while gender-based morphometric differences exist, their impact on block success appears minimal.

### 4.3. Morphology and Technical Challenges

We found that closed and irregular hiatus morphologies were associated with reduced hiatus width and canal depth, features likely to complicate caudal access. Abera et al. (2021) examined dry human sacra and reported significant morphological and morphometrical variability in the sacral hiatus, emphasizing its potential implications for caudal epidural block success [5]. Patra et al. (2024) analyzed 250 CT cases and noted that morphologic variations directly influence procedural feasibility [14]. Nastoulis et al. examined 200 dry sacra and further emphasized the relevance of morphometric thresholds for procedural success [7]. Together, these findings highlight the roles of morphology as a determinant of technical complexity during a caudal block.

### 4.4. ROC Analysis and Predictive Thresholds

ROC analysis identified a hiatus width a threshold of <8 mm as being predictive of block failure, yielding an AUC of 1.00 (95% CI: 0.98–1.00). Although this result indicates excellent discriminatory performance, such perfect results are exceptionally uncommon in clinical research and most likely reflect derivation-cohort bias and the study’s retrospective design. Internal validation using bootstrap resampling (AUC = 0.99) and repeated cross-validation (AUC = 0.98) confirmed the consistency of the finding, yet the risk of overfitting remains considerable. As highlighted by Babyak [15] and Steyerberg et al. [16], overfitted models often produce overly optimistic estimates that cannot be generalized to independent datasets. Therefore, this threshold should be regarded as exploratory and hypothesis-generating, warranting confirmation through external, multicenter validation before clinical application.

### 4.5. Clinical Utility and Decision Curve Analysis

Decision curve analysis suggested that applying the <8 mm threshold may provide a net benefit across threshold probabilities of 0.2–0.6, indicating possible clinical utility. However, Vickers [17] has highlighted that decision curve analysis is exploratory unless supported by external validation. Thus, while our DCA suggests clinical relevance, its application should be interpreted cautiously.

### 4.6. Comparisons with the Literature

Several recent studies have confirmed the variability of sacral hiatus morphometry across populations. Rafeeq et al. examined 150 dry sacra from Indian individuals and reported population-specific morphometric thresholds for caudal anesthesia [18]. Kılıçaslan et al. (2015) performed a multidetector CT-based morphometric analysis of the sacral canal and hiatus, revealing substantial interindividual variation in the size and orientation of the hiatus relevant to caudal epidural access [12]. Yagi et al. analyzed 110 CT scans and revealed variations in coccygeal angulation between upright and supine positions [19]. Bagoji et al. analyzed 200 CT scans, Nastoulis et al. assessed 155 dry sacra and Patra et al. [7,13,14] studied 250 CT cases, all emphasizing the role of morphology and population-specific thresholds in caudal block success. Furthermore, emerging evidence from immuno-morphological investigations [20] and advanced MRI-based morphometric analyzes of the lumbosacral region (Rommelspacher et al., 2025) [21] highlights the progressive integration of anatomical characterization with image-guided procedural optimization. Collectively, these studies corroborate the variability observed in our cohort and underscore the importance of validating morphometric thresholds across imaging modalities and populations.

### 4.7. Clinical Implications

In practice, patients with a hiatus width <8 mm, an irregular or closed morphology, or a history of sacral trauma or surgery should be recognized as being at higher risk for caudal block failure. In such cases, alternative strategies—including angled needle approaches, trans-sacrococcygeal access, or the use of ultrasound or fluoroscopy—may be required. Recent clinical trials, such as Goel et al.’s involving 80 patients [22], Senkal & Sir’s involving 60 patients [23], Elashmawy et al.’s involving 90 patients [24], and Lee et al.’s involving 120 patients [25], have demonstrated that ultrasound and fluoroscopy guidance improve technical success rates. Tsai et al. (2022), in regard to a cohort of 70 patients [26], further reported that power Doppler ultrasonography enhances procedural accuracy. These findings collectively support the integration of imaging into procedural planning, particularly for patients with high-risk morphometry.

### 4.8. Limitations and Future Directions

The major limitations of this study include its retrospective, single-center design, the homogeneous population analyzed, and the lack of external validation. Given the lack of ethnic and regional diversity, our findings may not be generalizable to broader populations. Future studies should integrate multiethnic cohorts, different imaging modalities, and mechanistic analyses.

## 5. Conclusions

In conclusion, MRI-based morphometric evaluation of the sacral hiatus revealed that hiatus morphology and width may influence the technical success of a caudal epidural block. The observed AUC of 1.00 should be regarded as an exploratory, hypothesis-generating result, subject to a considerable risk of overfitting. This level should only be interpreted as a risk indicator rather than a definitive cutoff until validated in larger, heterogeneous populations. Our decision curve analysis indicated the potential clinical utility of MRI for predicting caudal epidural block success; however, external, prospective validation is indispensable before clinical application.

These findings are hypothesis-generating and must be confirmed in multicenter, prospective trials. Translating MRI-derived thresholds into ultrasound-based practice may further improve real-time decision-making and enhance procedural accuracy.

Clinicians should carefully evaluate patients with narrow (<8 mm) or atypical hiatus morphology and consider alternative strategies, including trans-sacrococcygeal access, angled needle insertion (~15°), or fluoroscopic/endoscopic guidance, particularly in technically challenging cases.

By integrating MRI-based morphometric assessment into procedural planning, interventional pain physicians may optimize clinical workflows, reduce technical failures, and enhance the safety and success of caudal epidural blocks.

## Figures and Tables

**Table 1:** Demographic characteristics of the participants (n = 240).

Variable	Mean ± SD	Median (IQR)	*p*-Value
Age (years)	51.1 ± 21.2	50.5 (30.8–70.2)	0.812
Height (cm)	165.1 ± 8.5	165.0 (158.8–173.0)	0.657
Weight (kg)	74.9 ± 14.4	74.5 (62.0–88.0)	0.702
BMI (kg/m^2^)	27.7 ± 6.2	26.9 (23.0–32.3)	0.590

Note: The mean age of the participants was 51.1 years, with equal sex distribution. Abbreviations: BMI, Body Mass Index; SD, Standard Deviation; IQR, Interquartile Range.

**Table 2:** Morphometric Parameters of the Sacral Hiatus.

Parameter	Mean ± SD	Median (IQR)	*p*-Value
Hiatus height (mm)	16.5 ± 7.4	16.3 (9.6–22.6)	0.212
Hiatus width (mm)	9.9 ± 3.0	9.6 (7.1–12.7)	0.103
Canal depth (mm)	5.9 ± 2.2	5.8 (4.1–7.7)	0.187
Sacrococcygeal angle (°)	40.8 ± 11.1	41.5 (31.1–49.5)	0.299

Note: The morphometric parameters demonstrated wide variability. Abbreviations: SD, Standard Deviation; IQR, Interquartile Range.

**Table 3:** Sacral hiatus morphometry according to sex.

Parameter	Female (n = 120)	Male (n = 120)	*p*-Value
Hiatus height (mm)	16.97 ± 7.25	15.99 ± 7.55	0.041
Hiatus width (mm)	9.92 ± 2.95	9.79 ± 3.06	0.048
Canal depth (mm)	5.68 ± 2.19	6.09 ± 2.22	0.121
Sacrococcygeal angle (°)	40.76 ± 11.42	40.93 ± 10.79	0.877

Note: Males tended to have slightly deeper canals.

**Table 4:** Sacral hiatus parameters according to morphology type.

Morphology	Height (mm)	Width (mm)	Depth (mm)	Sacrococcygeal Angle (°)
Irregular	16.52 ± 7.10	10.22 ± 3.05	5.92 ± 2.15	38.19 ± 10.76
U	15.89 ± 7.53	10.17 ± 3.13	5.99 ± 2.44	42.59 ± 10.90
V	17.38 ± 7.09	9.34 ± 2.82	5.64 ± 2.01	41.47 ± 10.15
Closed	16.27 ± 7.90	9.63 ± 2.96	5.96 ± 2.23	41.23 ± 12.05

Note: Width and depth were lower in closed and irregular types.

**Table 5:** Correlation of age and BMI with sacral hiatus parameters (Spearman).

Parameter	Correlation with Age (r, *p*)	Correlation with BMI (r, *p*)
Hiatus height (mm)	0.09 (*p* = 0.150)	0.03 (*p* = 0.607)
Hiatus width (mm)	−0.02 (*p* = 0.813)	−0.08 (*p* = 0.205)
Canal depth (mm)	−0.09 (*p* = 0.177)	0.03 (*p* = 0.683)
Sacrococcygeal angle (°)	0.11 (*p* = 0.099)	0.08 (*p* = 0.199)

Note: No significant correlations were found between age, BMI, and hiatus parameters. Abbreviations: BMI, Body Mass Index.

**Table 6:** Multivariate linear regression analyses.

Dependent Variable	Independent Variables	Model R^2^	Significance
Hiatus height	Age, Sex, BMI	0.08	all *p* > 0.05
Hiatus width	Age, Sex, BMI	0.06	all *p* > 0.05
Canal depth	Age, Sex, BMI	0.07	all *p* > 0.05

Note: Demographic variables did not significantly influence hiatus measurements. Abbreviations: BMI, Body Mass Index.

**Table 7:** Predictive value of hiatus width with respect to caudal block success (determined via ROC analysis).

Threshold (mm)	AUC	95% CI	Sensitivity (%)	Specificity (%)
<8	1.00	0.98–1.00	100	100

Note: AUC values are based on internal validation only and may be optimistic without external validation. Abbreviations: AUC, Area Under the Curve; CI, Confidence Interval.

## Data Availability

The data supporting the findings of this study are available from the corresponding author upon reasonable request. Due to institutional regulations, the raw data cannot be made publicly available.

## Abbreviations

AUC	Area Under the Curve
BMI	Body Mass Index
CI	Confidence Interval
CT	Computed Tomography
DCA	Decision Curve Analysis
HL test	Hosmer–Lemeshow Test
ICC	Intraclass Correlation Coefficient
IQR	Interquartile Range
MPR	Multiplanar Reformatting
MRI	Magnetic Resonance Imaging
OR	Odds Ratio
PACS	Picture Archiving and Communication System
ROC	Receiver Operating Characteristic
SD	Standard Deviation
SPSS	Statistical Package for the Social Sciences
STROBE	Strengthening the Reporting of Observational Studies in Epidemiology
TSE/SPACE	Turbo Spin Echo/Sampling Perfection with Application optimized Contrasts using Different Flip Angle Evolution
VIF	Variance Inflation Factor
